# High heterogeneity in community-acquired pneumonia inclusion criteria: does this impact on the validity of the results of randomized controlled trials?

**DOI:** 10.1186/s12879-018-3515-9

**Published:** 2018-12-03

**Authors:** Clara Flateau, Josselin Le Bel, Sarah Tubiana, François-Xavier Blanc, Christophe Choquet, Blandine Rammaert, Patrick Ray, Christophe Rapp, Cécile Ficko, Catherine Leport, Yann-Erick Claessens, Xavier Duval, on behalf of the ESCAPED study group, Y. E. Claessens, X. Duval, E. Bouvard, M. F. Carette, M. P. Debray, C. Mayaud, C. Leport, N. Houhou, S. Tubiana, M. Benjoar, F. X. Blanc, A. L. Brun, L. Epelboin, C. Ficko, A. Khalil, H. Lefloch, J. M. Naccache, B. Rammaert, A. Abry, J. C. Allo, S. Andre, C. Andreotti, N. Baarir, M. Bendahou, L. Benlafia, J. Bernard, A. Berthoumieu, M. E. Billemont, J. Bokobza, E. Burggraff, P. Canavaggio, E. Casalino, S. Castro, C. Choquet, H. Clément, L. Colosi, A. Dabreteau, S. Damelincourt, S. Dautheville, M. Delay, S. Delerme, L. Depierre, F. Djamouri, F. Dumas, M. R. S. Fadel, A. Feydey, Y. Freund, L. Garcia, H. Goulet, P. Hausfater, E. Ilic-Habensus, M. O. Josse, J. Kansao, Y. Kieffer, F. Lecomte, K. Lemkarane, P. Madonna, O. Meyniard, L. Mzabi, D. Pariente, J. Pernet, F. Perruche, J. M. Piquet, R. Ranerison, P. Ray, F. Renai, E. Rouff, D. Saget, K. Saïdi, G. Sauvin, E. Trabattoni, N. Trimech, C. Auger, B. Pasquet, S. Tamazirt, J. M. Treluyer, F. Tubach, J. Wang, O. Chassany, C. Misse

**Affiliations:** 1 Service de Maladies Infectieuses, Centre Hospitalier de Melun, Melun, France; 2 Département de Médecine Générale, Université Paris Diderot, Sorbonne Paris Cité, 75018 Paris, France; 3 Université Paris Diderot, Sorbonne Paris Cité, Paris, France; 4 IAME – UMR 1137, Université Paris Diderot, Paris, France; 5 Inserm CIC 1425, Paris, France; 6 Hôpital Bichat Claude Bernard, Assistance Publique des Hôpitaux de Paris, Paris, France; 7 Service de Pneumologie, Institut du Thorax, CHU Nantes, Nantes, France; 8 Inserm, UMR1087, Institut du Thorax, Nantes, France; 9 CNRS, UMR 6291, Nantes, France; 10 Université de Nantes, Nantes, France; 11 Service d’Accueil des Urgences, Hôpital Bichat Claude Bernard, Paris, France; 12 Service de Maladies Infectieuses et Tropicales, CHU de Poitiers, Université de Poitiers, Poitiers, France; 13 Inserm U1070, Poitiers, France; 14 Service d’Accueil des Urgences, Hôpital Tenon, Hôpitaux Universitaires Est Parisien, Assistance Publique des Hôpitaux de Paris, Paris, France; 15 DHU “Fighting against ageing and stress” (FAST), UPMC Paris 6, Paris, France; 16 Service des Maladies Infectieuses, Hôpital d’Instruction des Armées Bégin, Saint-Mandé, France; 17 Unité de Coordination du Risque Epidémique et Biologique (COREB), Assistance-Publique Hôpitaux de Paris, Paris, France; 18 Department of Emergency Medicine, Centre Hospitalier Princesse Grace, Principality of Monaco, France

**Keywords:** Inclusion criteria, Clinical trial, Randomized clinical trial, Diagnostic criteria, Community- acquired pneumonia

## Abstract

**Background:**

There is no consensus on the most accurate combination of diagnostic criteria to define community acquired pneumonia (CAP). We describe inclusion criteria in randomized controlled trials (RCT) of CAP and assess their performance for the diagnosis of formally identified CAP.

**Methods:**

RCTs related to CAP recorded on ClinicalTrials.gov were analysed. Due to high heterogeneity, we divided close CAP inclusion criteria into patterns (i.e. combinations of inclusion criteria). To assess their diagnostic performances, these CAP definition patterns were applied to a reference population of 319 suspected CAP patients, in whom the CAP diagnosis had been confirmed (*n* = 163) or excluded (*n* = 156) by an adjudication committee after a systematic thoracic CT-scan and a 28-day follow-up period.

**Results:**

In the 47 RCTs included in the analysis, 42 different CAP inclusion criteria combinations were identified and 8 patterns created. This heterogeneity was not explained either by the trials’ methodology or by their objectives. When applied to the reference population, the performance ranges of the 8 definition patterns were 9.8–56.4% for sensitivities, 56.4 97.4% for specificities, 63.6 83.6% for positive predictive values and 50.8–66.7% for negative predictive values. None of the CAP definitions had both sensitivity and specificity superior to 65%. Depending on the CAP definition, the rate of included patients without CAP (“false positives”) ranged from 1 to 21%.

**Conclusions:**

CAP diagnostic criteria within RCTs are heterogeneous, which may have far-reaching consequences on validity of RCT results.

## Background

Community-acquired pneumonia (CAP) is a leading cause of morbidity and the first cause of mortality by infectious disease in the European region. Approximately 3 million cases of CAP are reported annually in Europe, of which one-third are hospitalized [1]. Infectious pneumonia corresponds to the multiplication of microorganisms in alveoli responsible for a combination of non-specific pulmonary and general symptoms, with numerous alternative diagnoses [2]. Due to the deep localization of the infection, microbiological data, which may help to establish a diagnosis, is reported in less than 50% of patients and the diagnosis and aetiology frequently remains uncertain [3]. Thoracic-CT scan, which has proved its efficacy in confirming or invalidating the diagnosis of CAP, is probably to date the best non-invasive tool to confirm the diagnosis of CAP [4–6]. However, its cost and limited availability compared to chest X-ray prevent its widespread use as a diagnostic criterion.

Due to heterogeneity of clinical presentation, there are to date neither universal diagnostic criteria to define CAP, nor a validated diagnostic classification. Consequently, CAP definitions differ according to country, medical specialty and practice guidelines [7–9]. However, the lack of universal CAP diagnostic criteria might have consequences for clinical practice, epidemiological analyses, and also validity of randomized controlled trials (RCTs). In this context, a true and powerful evaluation of an intervention obviously requires the inclusion of a sufficient number of individuals truly presenting the targeted disease and representative of the entire infected population. This is even more true in non-inferiority trials, a frequently-used methodology in CAP RCTs [10], in which the inclusion of patients without the targeted disease might result in inaccurate estimation of the difference between arms of studies and an incorrect conclusion of non-inferiority of the evaluated intervention. Therefore, an assessment of existing diagnostic criteria for CAP is essential for clinical research.

In the present study, our principal objective was to assess the heterogeneity of CAP diagnostic criteria used in RCTs and to identify different patterns of CAP inclusion criteria. Furthermore, from the results of a previously-conducted study [4], establishing the diagnosis of CAP based on all currently available data and also on early systematic thoracic CT-scan, we assessed performance (i.e. sensitivity, specificity, positive predictive value and negative predictive value, likelihood ratios) of different CAP inclusion criteria patterns when applied to our reference population [4]*.*

## Methods

### Selection and data extraction from randomized controlled trials

We searched ClinicalTrials.gov using the key words « community-acquired pneumonia » (last connection on October 1st 2018). The eligible trials were RCTs including adults with CAP independent of the trials’ primary objective. Trials including a paediatric population, severe CAP and those withdrawn before enrolment were excluded.

The following data were extracted from the ClinicalTrials.gov website: Clinical Trial Number (NCT); declaration, start and completion dates; type of sponsor (industrial or academic); study design; primary objective of the study; extensive list of inclusion criteria (including the CAP diagnostic criteria); presence of a CAP severity score (Pneumonia Severity Index or CURB 65) and the list of exclusion criteria. The study design included the number of centres, national or international recruitment, the location of inclusion (community or hospital), the blinding method (simple/double-blind or open-label trial), the superiority or non-inferiority design, and the field of the study (evaluation of diagnostic criteria, evaluation of biomarkers, choice or duration of antibiotic treatment). Data collection was performed independently by two investigators (JLB and CF). Disagreements were resolved by consensus.

We combined different methods to collect CAP diagnostic criteria. First, we collected data available on the ClinicalTrials.gov declaration. We also systematically contacted the investigators by email to confirm and complete their CAP diagnostic criteria. A second e-mail was sent 1 month later to the non-responders. Finally, when CAP diagnostic criteria were not available, we searched PubMed for publications and looked for CAP diagnostic criteria in the full article, when available.

### Diagnostic criteria for CAP

For each trial, criteria used to establish CAP diagnosis were collected. To allow the comparison, these criteria were categorized as 1) respiratory symptoms (dyspnea, chest pain, cough, sputum), 2) pulmonary auscultation abnormalities, 3) general symptoms (fever, malaise, chills), 4) biological criteria (leucocytosis, C-reactive protein increase, procalcitonin increase) and 5) thoracic radiological criteria.

For each trial, we specified which criteria were mandatory or optional to establish the CAP diagnosis, and their combination*.* As there were numerous combinations of mandatory and optional criteria to define CAP (and therefore to include CAP patients), we gathered CAP definitions with quite similar criteria. In this report, we will use the term “CAP definition patterns” to refer to these different patterns of CAP inclusion criteria.

### Performance of CAP definition patterns to properly identify patients with CAP in a reference population

#### Reference population

To assess the performances of each CAP definition pattern, the different CAP definition patterns were applied to the “ESCAPED” database. ESCAPED is a prospective, multicenter, interventional study which assessed the impact of a systematic thoracic CT-scan on the diagnosis of CAP in patients visiting the emergency department for a suspected CAP [4]. Patients were included in the database if 1) they presented at least one symptom of systemic infection (temperature > 38 °C or < 36 °C, heart rate > 90/min, respiratory rate >  20/min) AND 2) one recent respiratory symptom (cough, lateral chest pain, sputum, dyspnea, localized crackles) AND 3) had had a chest radiography AND 4) the clinician suspected CAP. The inclusion criteria did not include chest X-ray infiltrate, which made possible the inclusion of true CAP without infiltrate on chest X-ray, for which pulmonary involvement was secondarily established by thoracic CT-scan.

For each of the 319 included patients, an adjudication committee composed of a senior specialist in radiology, pneumonology and infectious diseases established the diagnostic probabilities of CAP according to a 4-level Likert scale (definite; probable; possible; excluded) from all available data at day 28 post-diagnosis. Based on the evaluation of the adjudication committee, there were 150 definite CAP cases, 13 probable CAP cases, 34 possible CAP cases, and 122 excluded CAP cases [4]. For the present study, we grouped definite and probable CAP in a single category and considered 163 patients as having CAP, compared to 156 who were considered as not having CAP.

#### Performances of CAP definition patterns

The comprehensiveness of clinical, biological and radiological data collected in the ESCAPED database, including all of the inclusion criteria used in the RCTs identified in the literature, allowed us to apply each CAP definition pattern to this database. For each CAP definition pattern, we aimed to determine if it accurately identified patients with CAP from the reference population of the ESCAPED database. We determined, among the 319 patients of the ESCAPED database, the number of patients who would have been included or excluded in the RCT, according to each CAP definition pattern. For each patient, we already knew if he was considered as having “definite or probable CAP”, and “excluded or possible CAP” by the adjudication committee.

This resulted, for each CAP definition pattern evaluated, in a number of “true positives” patients (i.e. considered as having CAP by the evaluated CAP definition pattern and by the adjudication committee), a number of “false positive” patients (i.e. considered as having CAP by the evaluated CAP definition pattern but not by the adjudication committee), a number of “true negative” patients (i.e. considered as not having CAP by the evaluated CAP definition pattern and by the adjudication committee) and a number of “false negative” patients (i.e. considered as not having CAP by the evaluated CAP definition pattern but as having CAP by the adjudication committee).

### Statistical analysis

For each CAP definition pattern, after determination of the number of “true positive”, “true negative”, “false positive” and “false negative” patients, we calculated sensitivity, specificity, positive predictive value (PPV), negative predictive value (NPV), and positive and negative likelihood ratios for the diagnostic of CAP. All tests were two-sided, and *p*-values below 0.05 were considered to denote statistical significance. All statistical analyses were performed with SAS software (version 9.3).

## Results

### Randomized controlled trials selection and characteristics

**Fig. 1:**
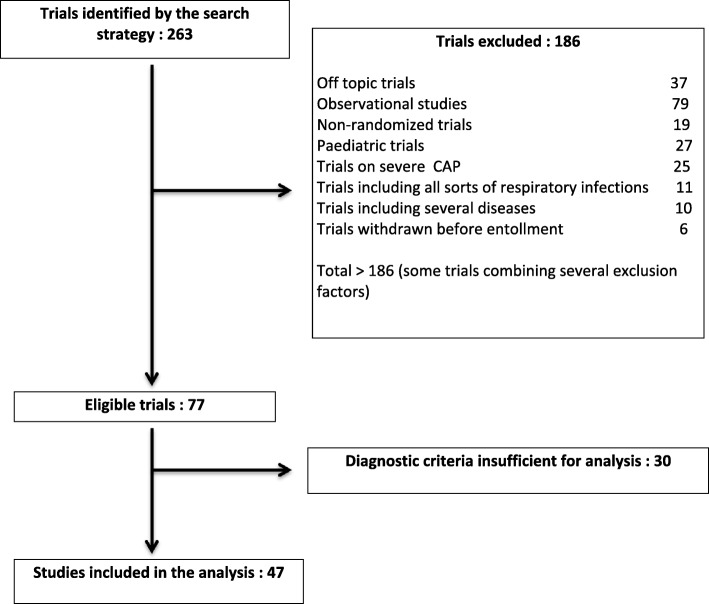
Selection of trials

The search yielded 263 trials, 77 of which met the inclusion criteria for the present study. Reasons for exclusion are shown in Fig. 1. The full diagnostic criteria for CAP were obtained in 47 out of the 77 (61%) trials; these 47 trials were included in the analysis: in 32/47 trials (68%), diagnostic criteria were correctly described in ClinicalTrials.gov database; in 8 (17%) they were obtained after contacting the investigator, and in 7 trials (15%), they were obtained after analysis of the published articles.

The 47 trials had been registered on ClinicalTrials.gov between 2002 and 2018. Seventeen trials (36%) had published results. The sponsor was industry in 64% of cases and academic in 36%. Most trials were double-blinded (74%) and the type of trial was specified in 58% of cases (non-inferiority 49%, superiority 9%). The objective of the trial was the evaluation of antibiotic choice in 32 (68%), of different antibiotic durations in 5 (11%), of other therapeutic strategies in 9 (19%), and of biomarkers in 3 (6%); some studies combined two of these objectives (Table 1).

**Table 1:** Characteristics of the trials included in the analysis (*N* = 47)

Characteristics	No of trials	%
Sponsor		
Industry	30	64
Academic	17	36
Number of centres		
Monocentric	5	11
Multicentric	38	81
Not specified	4	9
Country		
National	21	45
International	20	43
Not specified	6	13
Location		
Community	8	17
Hospital	31	66
Community and hospital	1	2
Blinding of the trial		
Simple blind	2	4
Double blind	35	74
Open-label	10	21
Type of trial		
Non-inferiority	23	49
Superiority	4	9
Not specified	20	43
Objective of the trial		
Antibiotic choice	32	68
Antibiotic duration	5	11
Other therapeutic strategy	9	19
Biomarkers evaluation	3	6
Severity score required for inclusion		
No	27	57
PSI^a^	18	38
CURB 65	2	4

^a^*PSI* Pneumonia severity index

### Diagnostic criteria for CAP

The search identified 42 different combinations of CAP diagnostic criteria in the 47 trials. In 44 out of the 47 trials (94%), a pulmonary infiltrate compatible with pneumonia on chest X-ray was mandatory for CAP diagnosis, while it had to be new in 38 trials. In one trial, it was an optional diagnostic criterion. In the two remaining trials, the chest X-ray result was not a diagnostic criterion.

The list and the combinations of the criteria were highly heterogeneous. Table 2 presents the frequency of each criterion (optional or mandatory) in the 47 trials. Fever was mentioned as an inclusion criterion in 98% of trials but was mandatory in 19%. At least one biological criterion was mentioned in 64% of trials and mandatory in 6%: mostly leukocytosis or C-reactive protein. According to the list of criteria and their 42 different combinations, eight CAP definition patterns were generated (Table 3). The CAP definition patterns frequently used were pattern 5 (infiltrate on chest X-ray and ≥ 1 or ≥ 2 criteria among fever, respiratory symptom and biological inflammatory syndrome, *N* = 19) and pattern 3 (infiltrate on chest X-ray and ≥ 1 respiratory symptom, *N* = 11). Definition patterns 6 and 7 had exclusively clinical diagnostic criteria. There was no correlation between the characteristics of the trials (investigator, location, number of centers, design and objectives) and one specific CAP definition pattern. Among the 21 non-inferiority trials, the combinations of diagnostic criteria for CAP belonged to CAP definition patterns 2 (*N* = 3), 3 (*N* = 9), 4 (*N* = 5), 5 (*N* = 4) and 8 (*N* = 1).

**Table 2:** Frequency of the pneumonia diagnostic criteria in the 47 trials

Criteria	CAP diagnostic criteria			
	Cited		Mandatory	
	N	%	N	%
At least one respiratory sign or symptom	47	100	25	53
Dyspnoea or polypnea	40	85	2	4
Chest pain	22	47	1	2
Cough	44	94	4	9
Purulent sputum	30	64	2	4
Sputum	18	38	3	6
At least one pulmonary auscultation abnormality	40	85	3	6
Rales	24	51		
Crackles	10	21		
Pulmonary consolidation	26	55		
Other abnormality	10	21		
Unspecified	10	21		
Focal abnormality	2	4		
General symptoms: fever	46	98	9	17
At least one biological abnormality	30	64	3	6
Leucocytosis	23	49		
C-reactive protein increase	5	11		
Procalcitonin increase	1	2		
*Streptococcus pneumoniae* antigenuria	1	2		
Pulmonary infiltrate compatible with CAP^a^ on chest X-ray	45	96	44	94
Recent infiltrate	38	81	38	81
Lobar or multilobar infiltrate	5	11	5	11

^a^*CAP* Community-acquired pneumonia

**Table 3:** Community-acquired pneumonia definition patterns of the 47 trials according to the characteristics of the trials

CAP definition pattern		Number of trials	Type of trial		
			Non-inferiority	Superiority	NS
1	Infiltrate on chest X-ray	3	–	1	2
	*and* ≥ 1 respiratory symptom				
	*and* fever				
	*and* biological inflammatory sd				
2	Infiltrate on chest X-ray	3	3	–	–
	*and* ≥ 1 respiratory symptom				
	*and* fever				
3	Infiltrate on chest X-ray	11	9	–	2
	*and* ≥ 1 respiratory symptom				
4	Infiltrate on chest X-ray	8	5	1	2
	*and* ≥ 2 respiratory symptoms				
5	Infiltrate on chest X-ray	19	4	2	13
	*and* ≥ 1 criterion / > 2 criteria among				
	-*fever*				
	-*respiratory symptom*				
	-*biological inflammatory sd*				
6	Fever	1	–	–	1
	*and* dyspnoea / polypnoea				
	*and* new cough				
	*and* purulent sputum				
	*and* abnormal pulmonary auscultation				
7	Fever	1	–	–	1
	*and* new sputum				
	*and* ≥ 2 criteria among				
	-*dyspnoea*				
	-*polypnoea*				
	-*chest pain*				
8	Infiltrate on chest X-ray	1	1	–	–
	*and* ≥ 4 criteria among				
	-*fever*				
	-*respiratory symptoms*				

*NS* Type of study not specified, *AB* Antibiotic, *sd* Syndrome, *CAP* Community-acquired pneumonia

### CAP definition patterns’ diagnostic performance

**Fig. 2:**
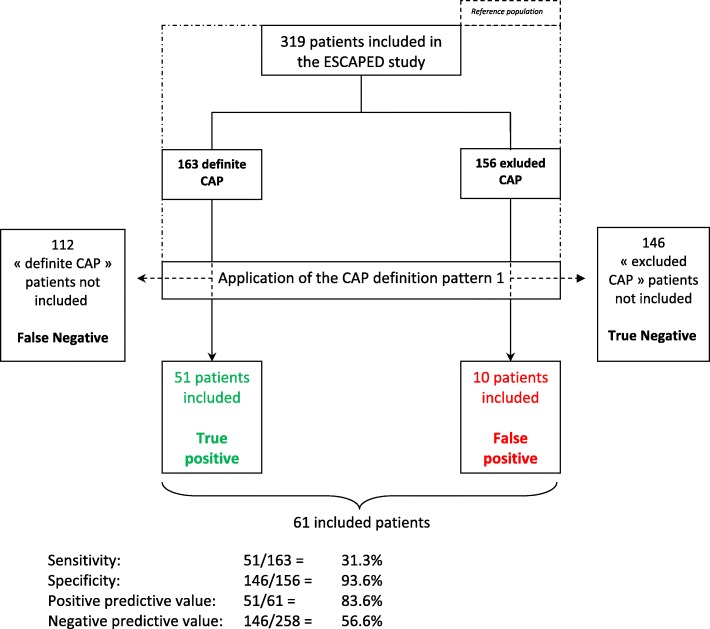
Application of the CAP definition patterns to the 319 patients of the ESCAPED database, example of CAP definition pattern 1

The performances of the 8 CAP definition patterns applied to the ESCAPED database are presented in Fig. 2 and Table 4. For example, considering CAP definition pattern 1, its application to the 319 ESCAPED patients would have led to the inclusion of 61 of the 319 patients. Among these 61 patients, 51 had definite CAP according to the adjudication committee and were therefore “true positives”, while the 10 other patients included by CAP definition pattern 1 were actually “false positives”. CAP definition pattern 1 failed to include 112 patients with CAP (“false-negatives”). Finally, the 146 other ESCAPED patients were correctly identified by CAP definition pattern 1 as patients without CAP (“true negatives”). This allowed the calculation of a sensitivity of 31.3%, a specificity of 93.6%, a PPV of 83.6%, a NPV of 55.6%, a positive likelihood ratio of 4.88 and a negative likelihood ratio of 0.73 for the diagnosis of CAP by CAP definition pattern 1 (Fig. 2).

**Table 4:** Performances of the eight CAP definition patterns applied to the 319 patients of the ESCAPED database for the diagnosis of CAP

CAP definition pattern	Number of included patients among the 319 Escaped patients	True Positive	False Negative	False Positive	True Negative	Sensitivity (%)	Specificity (%)	Positive Predictive Value (%)	Negative Predictive Value (%)	Positive likelihood ratio	Negative likelihood ratio
1	61	51	112	10	146	31.3	93.6	83.6	55.6	4.88	0.73
2	103	77	86	26	130	47.2	83.3	74.8	60.2	2.83	0.63
3	187	119	44	68	88	73.0	56.4	63.6	66.7	1.67	0.48
4	170	110	53	60	96	67.5	61.5	64.7	64.4	1.75	0.53
5	178	116	47	62	94	71.2	60.3	65.2	66.7	1.79	0.48
6	20	16	147	4	152	9.8	97.4	80.0	50.8	3.84	0.93
7	50	38	125	12	144	23.3	92.3	76.0	53.5	3.03	0.83
8	103	71	92	32	124	43.6	79.5	68.9	57.4	2.12	0.71

The CAP definition patterns 6 and 7, exclusively based on clinical criteria, were very specific (97.4 and 92.3%) but had low sensitivities (9.8 and 23.3%, respectively). Among the 5 other CAP definition patterns including chest X-ray in diagnostic criteria, patterns 3, 4, and 5 maximized sensitivity (from 67.5 to 73%) whereas patterns 1 and 2 maximized specificity (from 83.3 to 93.6%). The most sensitive CAP definition patterns (patterns 3, 4 and 5) were the most used in RCTs (Table 3). Overall, none of the CAP definition patterns obtained both sensitivity and specificity superior to 65%. Among the six CAP definition patterns including chest X-ray, the number of “false-positive” among the included population varied by a factor of 6 (from 10 for definition pattern 1 to 68 for definition pattern 3).

## Discussion

In this study, we report the considerable heterogeneity of CAP inclusion criteria in RCTs and, by applying these criteria to a reference population, explored through systematic CT-scan, we underline the potential risk of inclusion of patients without CAP, in RCTs focused on CAP.

We show that the heterogeneity of CAP inclusion criteria applies to the number of criteria, their type (pulmonary or general symptoms, biological criteria, radiological abnormalities), their optional or mandatory nature, their multiple combinations, and was not explained by the characteristics of the trials, their objectives, their methodology or their sponsor.

When applied to a unique reference population of patients suspected to have CAP visiting the emergency department, this variety of CAP definition patterns resulted in variations of the number of patients identified as having CAP and their diagnostic authenticity. Furthermore it affected the proportion of patients with or without definite CAP who would be included in RCTs using these inclusion criteria (variation by a factor of 6 of the number of false positive patients). This variability in the included population has implications in terms of the trials’ internal and external validities. Indeed, the evaluation of therapy in individuals who do not have the disease negatively impacts the internal validity of both non-inferiority and superiority trials. Considering non-inferiority trials (the majority of those analysed), factors that result in smaller differences between study groups will lead to the false conclusion that the new treatment is not inferior to the gold standard [11–13]. This is particularly true for CAP definition patterns with a high rate of false positive CAP. Considering superiority trials, including patients without CAP would result in a loss of statistical power to demonstrate the superiority of a potentially effective therapy.

This heterogeneity in CAP definitions also calls into question the external validity (application of the results) and furthermore prevents future comparisons of these different trial results which, even if they share the same objective and the same declared recruited individuals (CAP patients), will draw conclusions from populations with non-comparable characteristics. This may very well also explain why conclusions of different CAP RCTs differ so much in the literature [13], and limited the conclusions based on the metaanalysis of such studies.

Our study has some limitations. First, we did not test the performance of each of the 42 combinations of diagnostic criteria in the database. We grouped them into eight CAP definition patterns for analysis, which might have resulted in an under-estimation of the heterogeneity of the diagnostic performance of the diverse CAP definitions. Second, the choice of our reference population may have biased the analysis. However, the choice of a reference population to evaluate the performance of diagnostic criteria inevitably results in a selection bias. We believe that the ESCAPED population is the more acceptable reference population, as data collected in the database included all the diagnostic criteria of the eight CAP definition patterns, and the adjudication committee based its judgment on all available data including a thoracic CT-scan and a 28-day follow-up. In this population, 27% of the CAP diagnoses established based on the existence of an infiltrate on chest X-ray were finally excluded by thoracic CT-scan evaluation [4]. Third, we considered in our analysis that “confirmed” and the “probable” CAP patients based on the adjudication committee classification had definite CAP. However, considering only confirmed CAP patients as definite CAP gave similar results (data not shown). Finally, we based our definition of true CAP considering CT-scan as the adequate gold standard for the diagnosis of CAP. It remains to date the most useful non-invasive technique to establish and/or exclude pneumonia diagnosis, [6].

The question arises whether our results allow the identification of the most accurate combination of inclusion criteria for RCTs. The CAP definition patterns 1, 6 and 7, provided excellent specificity but their low sensitivity prevents their common use in clinical research. The three most sensitive CAP definition patterns – displaying quite similar performances - are already the most used in RCTs, attesting to the pragmatism of investigators. The issue is to improve their specificity. We suggest that the per-protocol analysis of any CAP trial should be performed on the sub-population of patients whose CAP diagnosis is established a posteriori by an adjudication committee, taking into account all available data, including microbiological results and follow-up, and if possible a thoracic CT-scan.

This “two-step” strategy would have the merit of being appropriate in every situation: when high sensitivity of the diagnosis is the priority in the context of urgent CAP care, as delay in antibiotic treatment is associated with poorer prognosis, but also in therapeutic trials, where more stringent inclusion criteria are needed. This strategy would increase the validity of both epidemiological studies and randomized clinical trial results, and make possible the comparison of the results.

## Conclusions

The inclusion criteria for CAP in RCTs are highly heterogeneous. Their diagnostic performances vary considerably and some definitions might lead to the inclusion of a proportion of patients without CAP or to missing patients with a CAP diagnosis. This heterogeneity may impact the interpretation of the results of randomized trials, which remain the basis of evidence-based guidelines. To “plagiarize” Carl Nathan [14], it makes no sense to use twenty-first century technology to develop drugs targeted at specific infections whose evaluation is based on RCTs which use nineteenth-century CAP diagnostic criteria. We suggest a “two-step strategy”, associating a sensitive combination of inclusion criteria identified in our study, with a systematic per-protocol analysis on the sub-population of patients whose CAP diagnosis is established a posteriori by an adjudication committee, including if possible a thoracic CT-scan.

## Abbreviations

CAP: Community-acquired pneumonia
NPV: Negative predictive value
PPV: Positive predictive value
PSI: Pneumonia severity index
RCT: Randomized controlled trial
